# Fecal Microbiota Transplantation for the Treatment of Inflammatory Bowel Disease: An Update

**DOI:** 10.3389/fphar.2020.574533

**Published:** 2020-09-18

**Authors:** Pufang Tan, Xiaogang Li, Jun Shen, Qi Feng

**Affiliations:** ^1^ Division of Gastroenterology and Hepatology, Baoshan Branch, Renji Hospital, School of Medicine, Shanghai Jiao Tong University, Shanghai, China; ^2^ Division of Gastroenterology and Hepatology, Key Laboratory of Gastroenterology and Hepatology, Ministry of Health, Inflammatory Bowel Disease Research Center, Renji Hospital, School of Medicine, Shanghai Jiao Tong University, Shanghai Institute of Digestive Disease, Shanghai, China; ^3^ Department of Radiology, Renji Hospital, School of Medicine, Shanghai Jiao Tong University, Shanghai, China

**Keywords:** fecal microbiota transplantation, inflammatory bowel disease, ulcerative colitis, Crohn’s disease, microbiome

## Abstract

Fecal microbiota transplantation (FMT) has successfully been applied for the treatment of recurrent *Clostridioides difficile* infection (CDI), which has led to studies on its application to other gastrointestinal diseases and extraintestinal diseases associated with gut microbiota dysbiosis. Recently, the results of FMT for patients with inflammatory bowel disease (IBD) have been encouraging. However, studies have not fully clarified the clinical application of this emerging therapy. Here, we aimed to review the current knowledge in this fast-growing field and characterize the effectiveness, safety and mechanisms of FMT for the treatment of IBD patients.

## Introduction

Fecal microbiota transplantation (FMT) refers to the therapeutic procedure of transplanting fecal bacteria from healthy persons into patients (Gupta and Khanna, 2017). FMT is aimed at restoring the colonic microbiota through the introduction of fit bacterial population by infusing the gut microbiota, for example *via* colonoscopy, the orogastric tube, enema, or orally in the form of a capsule that contains the freeze-dried substance, to obtain an intestinal microbiota from a suitable donor. This therapeutic method has been proven to be incredibly successful for the treatment of recurrent *Clostridioides difficile* infection (CDI). (Drekonja et al., 2015)

The techniques of FMT deal with three key aspects, including donor selection, together with preparation of donor substance, and FMT delivery (Kelly et al., 2015; Mullish et al., 2018). Appropriate donors should include those of the age range of 18–60 years (Anand et al., 2017; Mullish et al., 2018), in addition to those with a BMI from 18 to 30 kg/m^2^ (Alang and Kelly, 2015). The employment of multiple donors was more effective compared with single donor, because that multiple donors increase the microbial diversity in the fecal suspension compared with that of single donor (Levy and Allegretti, 2019). In each of the FMT preparations, the stool requires a weight of at least 25 g for lower gastrointestinal delivery and 12.5 g for upper gastrointestinal delivery (Cammarota et al., 2019). The final fecal substance preserved frozen at a temperature of −80°C is considered to have a maximum shelf life of 2 years (Cammarota et al., 2019). Prior to utilisation, the frozen fecal material should be thawed at 37°C, followed by use within a period of 6 h of thawing (Costello et al., 2015). The fecal microbiota could be supplied *via* the upper GI route (endoscopically or with the use of a nasogastric tube, nasoduodenal tube, or nasojejunal tube), the lower GI route (retention enema, colonoscopy), or through capsules (which contain either the freeze-dried or lyophilised fecal substance) (Mullish et al., 2018). Proton pump inhibitor and prokinetics (for example, metoclopramide) should be considered before FMT *via* the upper GI route (De Jager et al., 2012; Cohen et al., 2016). Bowel lavage must be performed before FMT *via* the lower GI route. Specifically, Polyethylene glycol preparations are preferred (Kelly et al., 2016). A single dose of loperamide (or other anti-exercise drugs) could be considered subsequent to the lower GI FMT delivery. Further, a minimum 24-h washing period is required from the last dose of antibiotics to FMT treatment (Cammarota et al., 2015). When recipients also show signs of long-term antibiotic use within 8 weeks after FMT, it is necessary to consult infectious disease experts or medical microbiologists for advice (Terveer et al., 2017). 

FMT is considered approximately 90% success rate for individuals with recurrent or refractory CDI (Quraishi et al., 2017). In 2013, FMT was approved by the USA Food and Drug Administration for clinical applications of recurrent or refractory CDI (Surawicz et al., 2013). In addition to CDI, there are some studies that have proven that FMT could emerge as a productive method to treat inflammatory bowel disease (IBD). IBD refers to an intestinal disorder, including ulcerative colitis (UC) as well as Crohn’s disease (CD). IBD is characterized by chronic inflammation of the gastrointestinal tract, as well as the periodicity of disease progression and remission. During disease activity, patients are likely to present with diarrhoea, coupled with nausea, loss of weight, anepithymia, fever, and celialgia (White et al., 2018). However, the precise pathophysiology is not entirely clear and the aetiology is multifactorial, influenced by individual genetic susceptibility, the external environment, internal gut microbiota, and the host immune response (Zhou et al., 2017). The imbalance of the gut microbiota has been suggested to markedly impact IBD progression (Zuo and Ng, 2018). Metabolomic and metagenomics research has identified the characteristics of IBD microbiota, in addition to finding a general decline in bacterial diversity, with a particular decline in members of *Lachnospiraceae*, as well as the *Bacteroidetes phylum*, coupled with an increase in *Proteobacteria* and *Actinobacteria* (Henson and Phalak, 2017). Biopsy samples attained from patients with CD were found a decline in *Fecalibacterium prausnitzii* which has the effects of anti-inflammatory (Sokol et al., 2008).

FMT is currently regarded as a productive intervention for recurring CDI (Bakken et al., 2011). Meanwhile, the use of FMT in IBD does not result in the same exceptional outcomes. However, emerging data have suggested the possibility that this therapy could have a better effect on these diseases. Reviewing the current evidence on the utility of FMT for the treatment of IBD, we focus on the effectiveness, safety and mechanisms of this new treatment, as well as ongoing research in this growing field.

## Efficacy and Safety of FMT for IBD

The traditional methodology of treating IBD was aimed at reducing the inflammatory. Nevertheless, the latest insights focus on the microbiome, in addition to the idea of dysbiosis and its latent role in IBD pathogenesis, resulting in irreplaceable therapeutic methodologies like FMT, a fascinating area of investigation. FMT was identified as a standard therapy for recurrent CDI, but its efficacy for IBD therapy is controversial (Basso et al., 2018). Most of the researches did not indicated key negative events or severe negative events that were considered medically associated with FMT treatment (Paramsothy et al., 2017b). Sood et al. (2020) performed a retrospective analysis of 101 active UC patients receiving multiple FMT *via* colonoscopy. The study found that the most common short-term adverse events of FMT included abdominal discomfort (30.8%), flatulence (15.9%), abdominal distension (9.8%), borborygmi (7.9%), and low-grade fever (7.6%). The long-term adverse events were arthritis/arthralgia (6.5%), urticaria (4.3%), depression (2.2%), allergic bronchitis (2.2%), and partial sensorineural hearing loss (2.2%). FMT appears to be a safe procedure.

### IBD With CDI

Patients who get IBD are easily infected by *C. difficile* (Rodemann et al., 2007). Khoruts et al. (2016) suggested that FMT could be successful for clearing CDI form both IBD and non-IBD patients. Nevertheless, in patients with underlying IBD, the efficiency of single FMT therapy for this application was lower than that in patients without IBD (74.4% *vs.* 92.1%; P = 0.0018). More than 1/4 of patients with IBD (25.6%) related to CDI experienced a significant flare of IBD after treatment with FMT. The latest study including 56 CDI patients (22 UC and 13 CD) who received FMT procedures *via* colonoscopy was successful for 48/56 (85.7%) of cases. In contrast, more than 50% of patients with UC experienced a sudden outbreak of IBD activity (Newman et al., 2017). The largest study of IBD patients with recurrent or refractory CDI included 67 patients (35 CD, 31 UC, and one indeterminate colitis). Respectively, the success rates of the first, second, and third fecal microbiota transplantation were 79%, 88%, and 90%. After FMT, 25 patients (37%) reported improvements in IBD disease activity, 20 patients (30%) had no change, and for nine patients (13%), disease worsened. Serious adverse events included CDI hospitalisation (2.9%), hospitalisation for IBD flares (2.9%), colectomy (1.4%), small bowel obstruction (1.4%), pancreatitis (1.4%), and cytomegalovirus colitis (1.4%) (Fischer et al., 2016). In conclusion, FMT has shown safety and efficacy in clearing CDI from patients with IBD, but the results need to be further evaluated systematically.

### Ulcerative Colitis

To date, there have been five published randomised controlled trials (RCT) that assessed the effectiveness of FMT in UC (**Table 1**) (Moayyedi et al., 2015; Rossen et al., 2015; Paramsothy et al., 2017a; Costello et al., 2019; Sood et al., 2019). In July 2015, the first research using a nasoduodenal tube for FMT found that there is no statistically significant difference in the rate of endoscopic and clinical remission between FMT and control groups (Rossen et al., 2015). However, the other four trials using lower gastrointestinal microbiota transplantation showed encouraging results (Moayyedi et al., 2015; Paramsothy et al., 2017a; Costello et al., 2019; Sood et al., 2019). Moayyedi et al. (2015) found that patients with UC for less than 1 year, but not longer, could enter a state of remission after treatment with FMT. These different findings are likely due to dissimilarities in the routes of administration, in addition to the stool donors and dosing schedules. Sood et al. (2019) conducted a study among 61 UC patients in clinical remission. Participants were randomly assigned to receive either FMT or placebo. Maintaining clinical remission at 48 weeks was achieved in 87.1% patients receiving FMT compared with 66.7% receiving placebo. There was a statistically significant impact of FMT on Endoscopic remission (FMT: 58.1% compared with placebo: 26.7%, p = 0.026) and histological remission (FMT: 45.2% compared with placebo: 16.7%, p = 0. 033). It is indicated that maintenance FMT in UC patients with clinical remission could help sustain endoscopic, histological, and clinical remission.

**Table 1:** Characteristics of randomized controlled trials on fecal microbiota transplantation (FMT) for ulcerative colitis.

Study	N (FMT/Control)	Disease activity	Control	Delivery	Frequency	Donor	Dosage	Primary endpoint	Follow up	Remission	Response
Rossen et al., 2015	48 (23/25)	SCCAI ≥ 4 and ≤ 11	Autologous fecal microbiota	Nasoduodenal	2 (weeks 0 and 3)	Single	Minimum 60 g stool in 500 ml saline	SCCAI ≤ 2 and ≥ 1 point decrease in Mayo endoscopic score	12 weeks	FMT 30.4%, control 20%, p= 0.51	FMT 47.8%, Control 52%
Moayyedi et al., 2015	75 (38/37)	Mayo Clinic score ≥ 4 with endoscopic score ≥ 1.	Water enema	Enema	6 (weekly)	Single	50 g stool in 300 ml water	Total Mayo score ≤ 2 with endoscopic score 0.	7 weeks	FMT 24%, control 5%, p=0.03*	FMT 39%, Control 24%, p = 0.16
Paramsothy et al., 2017a	81 (41/40)	Mayo Clinic score 4–10 with endoscopic score ≥ 1.	Saline enema	Initial colonoscopy then enema	40 (5/week for 8 weeks)	Pooled multi-donor	37.5 g stool in 150 ml	Total Mayo score ≤ 2, all subscores ≤ 1, and ≥ 1 point reduction in endoscopic score	8 weeks	FMT 27%, control 8%, p = 0.02*	FMT54%, Control 23%, p = 0.004*
Costello et al., 2019	73 (38/35)	Mayo Clinic score 310 with endoscopic score ≥2.	Autologous fecal microbiota	Initial colonoscopy then enema	3 (week 0 colonoscop-y, 2 enemas week 1)	Pooled multi-donor	Colonoscopy 50 g stool in 200 ml saline and glycerol, enema 25 g stool in 100 ml	Total Mayo score ≤ 2 and endoscopic score ≤ 1	8 weeks	FMT 32%, Contro l 9%, p = 0.02*	FMT 55%, Control 20%, p ≤ 0.01*
Sood et al., 2019	61(31/30)	Mayo score ≤ 2 with each subscore ≤ 1	NR	colonoscopic	7 (Weeks 0, 8, 16, 24, 32, 40, and 48)	Single and unrelated	100g in 200 ml	Mayo score ≤ 2, all subscores ≤ 1	48 weeks	FMT 87.1%, control 6.7%, p= 0.11	NR

SSCAI, simple clinical colitis activity index. *Statistically significant.

One recently published meta-analysis assessing the aforementioned four RCTs indicated that 39 of 140 (28%) patients achieved clinical remission in the FMT groups compared to 13 of 137 (9%) patients in the placebo groups, with an odds ratio of 3.67 (95% CI: 1.82–7.39, P < 0.01). Compared to 38 of 137 (28%) placebo patients, 69 of 140 (49%) patients in FMT groups achieved clinical responses, and the odds ratio was 2.48 (95% CI: 1.18–5.21, P = 0.02) (Costello et al., 2017). The latest systematic review and meta-analysis considered 27 research papers, which included 596 adult and paediatric IBD patients, and 459 patients were treated with FMT. During the follow-up period, 132 of 459 (28.8%) patients achieved clinical remission. The clinical effective rate was 53% (241/459). The overall clinical remission rate for UC was 21% (95% CI: 8–37%). As subgroup analyses revealed, the clinical remission rate prevailing in adult UC patients amounted to 26% (95% CI: 10%–48%), whereas paediatric patients showed a rate of 10% (95% CI: 0%–43%) (Fang et al., 2018). Hence, the therapeutic effect of FMT for patients with UC is very promising, particularly for patients with multiple transfusions through the lower digestive tract.

### Crohn’s Disease

The evidence on the effect of FMT in CD has recently been presented. There are currently several active trials studying the effectiveness of FMT for CD, and the results are diverse (**Table 2**) (Cui et al., 2015; Suskind et al., 2015; Wei et al., 2015; Vaughn et al., 2016; Vermeire et al., 2016; Goyal et al., 2018; Gutin et al., 2019; Sokol et al., 2020; Xiang et al., 2020). Xiang et al. (2020) studied 174 CD patients treated with FMT *via* Mid-gut including nasojejunal tube, endoscopy and mid-gut TET (except one through colonic TET). At 1 month after FMT, 76% (19/25), 72.7% (101/139), 70.6% (12/17) and 61.6% (90/146) of patients achieved improvement in hematochezia, abdominal pain, fever and diarrhoea respectively. At the final follow-up, the clinical remission was achieved in 20.1% (35/174) and the clinical response was achieved in 43.7% (76/174). Sokol and colleagues conducted a randomized controlled study of FMT in CD patients. Participants were randomly assigned either to the FMT group or the control group. The incidence of flare in the FMT group was lower than in the control group but there was not a statistically significant impact of FMT on clinical remission. The clinical remission at 10 and 24 weeks was 7/8 (87.5%) and 4/8 (50.0%) in the FMT group and 4/9 (44.4%) and 3/9 (33.3%) in the control group. Crohn’s Disease Endoscopic Index of Severity decreased at 6 weeks in the FMT group (p = 0.03) but not in the control group (p = 0.8). On the contrary, the CRP level increased at 6 weeks in the control group (p = 0.008) but not in the FMT group (p = 0.5) (Sokol et al., 2020).

**Table 2:** Characteristics of fecal microbiota transplantation for Crohn’s disease.

Study	N	Age (y)	Disease Activity	Delivery	Frequency	Donor	Dosage	Primary endpoint	Follow up	Remission	Response
Vaughn et al., 2016	19	> 18	HBI ≥ 5	Colonoscopy	1	Unrelated	50 g stool in 250 ml solution	HBI < 5 at 4weeks	26 weeks	53%	58%
Vermeire et al., 2016	6	28–53	Widespread involvement of the colon and/or ileum.	Nasojejunal or rectal tube	2 (daily for consecutive days)	Patient- directed donor	200 g stool in 400 ml saline	SES-CD < 3	8 weeks	0%	0%
Cui et al., 2015	30	15–71	HBI ≥ 7	Duodenum *via* gastroscope	1	Patient- directed donor	150– 200 ml	HBI ≤ 4	15 months	76.7%	86.7%
Wei et al., 2015	3	16–70	CDAI 150–400 and CRP > 10 mg/L	Nasojejunal or colonoscopy	1	Unrelated	60 g stool in 350 ml saline	CDAI < 150 and CRP < 10 mg/l	4 weeks	0%	NR
Goyal et al., 2018	4	2–22	PCDAI 10–40 or lactoferrin/calprotectin 2×upper limit of normal	Distal duodenum or jejunum *via* gastroscopy or colonoscopy	1	Patient- directed donor	150 g stool in 250– 300 ml saline	PDAI<10 or normalization of lactoferrin/calprotectin at 1 month	6 months	50%	75%
Suskind et al., 2015	9	12–19	PCDAI 10–29	Nasogastric tube	1	Related	30 g stool in 100– 200 ml saline	PCDAI < 10	12 weeks	78% at 2 weeks, 56% at 6 and 12 weeks	NR
Gutin et al., 2019	10	18-70	HBI ≥ 3	Colonoscopy	1	Unrelated donors	NR	decrease in HBI ≥ 3 at 1 month	12 months	10%	30%
Xiang et al., 2020	174	Median was 33 (IQR: 23–43)	The median of HBI was 8 (IQR: 6–10)	Mid-gut includ ing endoscopy, nasojejunal tube, and mid-gut TET (except one through colonic TET)	Median was 3.5 (IQR: 2–5)	Related and unrelated donors	NR	The rate of improvement in each therapeutic target	Median was 43 months (IQR: 28–59)	20.1%	43.7%
Sokol et al., 2020	17	18–70	HBI > 4	Colonoscopy	1	Unrelated donor	50–100g	successful colonization of donor microbiota	24 weeks	FMT group: 87.5% at 10 weeks; control group 44.4%	NR

HBI, Harwey Bradshaw Index; CDAI, Crohn’s Disease Activity Index; PCDAI, Pediatric Crohn’s Disease Activity Index; TET, transendoscopic enteral tubing; IQR, interquartile range; NR, not recorded.

Xiang et al. (2020) believed that FMT in CD against targeted therapeutics was efficient, especially hematochezia, abdominal pain, diarrhoea and fever. Whereas, Further research needs to be conduced to gain more high-quality data and provide conclusions with respect to the use of FMT for these patients and subsequently which CD phenotypes are most likely to benefit.

### Pouchitis

Pouchitis is the most common complication of patients with refractory UC with ileal pouch-anal anastomosis, and morbidity can reach nearly 50%. Similar to that with UC and CD, a decrease in intestinal microbial diversity plays a critical role in its pathogenesis. Antibiotics might induce remission of pouchitis but can lead to recurrence (Shen, 2012; Maharshak et al., 2017). Probiotics can prevent recurrence, which might be related to recovery of the mucosal barrier (Persborn et al., 2013). Five studies have been conducted to evaluate FMT in patient with pouchitis (**Table 3**) (Landy et al., 2015; Stallmach et al., 2016; Herfarth et al., 2019; Nishida et al., 2019; Selvig et al., 2020). In the four studies using a single-source fecal bacteria, none of the patients achieved remission of clinical symptoms (Landy et al., 2015; Herfarth et al., 2019; Nishida et al., 2019; Selvig et al., 2020). Stallmach et al. (2016) used multi-source fecal bacteria and many transplantations, and 80% of the patients achieved clinical remission, with the rest attaining a clinical response. It was lacked of available data and randomized controlled trials to demostrate the efficiency of FMT in treating Pouchitis. Recently, A prospective randomized controlled trial assessing the efficacy of FMT in pouchitis patients was prematurely stopped for low donor FMT engraftment (Selvig et al., 2020). Larger randomized controlled trials are needed to validate the effectiveness of FMT in pouchitis.

**Table 3:** Characteristics of fecal microbiota transplantation for pouchitis.

Study	N	Age (y)	Disease Activity	Delivery	Frequency	Donor	Dosage	Primary endpoint	Follow up	Remission	Response
Landy et al., 2015	8	24–63	Chronic antibiotic refractory Pouchetis,PDAI ≥ 7	Nasogastric	1	NR	18 g stool/30 ml saline solution)	PDAI ≤ 4	4 weeks	0%	25%
Nishida et al., 2019	3	24–52	PDAI ≥ 7	Colonoscopy	1	Related	NR	Reduction in total PDAI ≥ 3 at 8 weeks	8 weeks	0%	33%
Stallmach et al., 2016	5	26–40	Chronic antibiotic refractory pouchitis, PDAI 9–14	Jejunum *via* upper gastrointestinal tract endoscopy	1–7 (3–4 weeks intervals)	Two unrelated	75 g stool in 200 ml saline	NR	3 months	80% at 4 weeks, 60% at 3 months	100%
Herfarth et al., 2019	6	22–60	Chronic antibiotic refractory pouchi- tis (mPDAI≥5 and a history of ≥4 antibiotic therapies for pouchitis in the last 12 months)	Endoscopic (eFMT) and oral encapsulated (oFMT)	eFMT: 2; oFMT: daily for 14 days	Unrelated	eFMT: 12 g stool in 30 ml; oFMT: 4.2 g stool	The safety of the combined eFMT and oFMT	16 weeks	17%	17%
Selvig et al., 2020	19	18-74	Chronic pouchitis (pouch symptoms > 4 weeks and endoscopic evaluation con- frming infamma- tion of the pouch	Pouchoscopy	1–2	12 months	250 ml donor fecal suspen- sion (25 g stool)	Clinical improvement at week 4.	12 months	0	9% (among patients receiving double FMT)

PDAI, Pouchitis Disease Activity Index; FMT, fecal microbiota transplantation; eFMT, endoscopic FMT; oFMT, oral encapsulated FMT.

## Immunomodulatory Effects and Mechanisms of FMT

FMT therapy has been used for CDI for decades, whereas its use for IBD began since the year 2012. Reviewed from current researches, the immunomodulatory effects and mechanisms of FMT have been concluded:

### Intestinal Microbial Ecology

Various gastrointestinal disorders, which include IBD, are linked to changes in the intestinal microbiota composition and metabolic dysbiosis. It is not known if tissue impairment is caused by abnormal immune responses to normal microbiota or by a normal immune response to abnormal microbiota or if dysbiosis constitutes a cause or outcome of IBD (Sheehan et al., 2015; Ni et al., 2017). FMT is considered a promising therapeutic methodology for IBD patients, primarily to achieve outcomes of intestinal microbial restoration (Burrello et al., 2019).

FMT can increase the diversity of intestinal microorganisms, together with rebuilding the immune system and maintaining the balance of intestinal microecology. Even though comprehensive studies on increasing the microbial diversity of the intestines are continually being updated, many current studies could provide such proof-of-concept. Zeng et al. (2019) carried out a study analyzing the latest trials dealing with the immunomodulatory effects and underlying processes of probiotics and FMT, in addition to examining the effectiveness and safety of probiotics and FMT for medical experiments. Their group concluded that the intestinal microbiota from the donors limited intestinal permeability, inhibited intestinal epithelial cell apoptosis, re-established the function of the intestinal barrier, mitigated the production of proinflammatory cytokines and restored the metabolism of secondary bile acids in the intestinal tract. Further, FMT could compete with or antagonise pathogenic bacteria, in addition to enhancing insulin resistance. As a result, the patient’s immunity is improved. The IBD microbiome was discovered to promote inflammation and show signs of augmented oxidative stress, in addition to the enhanced secretion of type II toxins and the elevated level of virulence-associated bacterial genes (Erickson et al., 2012).

### T-Cell Populations

The populations of gut-linked immune cells make a critical contribution to initiating and sustaining intestinal inflammation, which occurs in patients with IBD and in experimental models of intestinal inflammation (Blumberg et al., 1999; Kaser et al., 2010). Natividad et al. (2015) showed that mice colonized with microbial populations from UC patients that were low in *Firmicutes* were more sensitive to colitis than mice colonized with *Firmicutes*-rich faeces or synthetic ecosystems. Here, *Firmicutes* bacteria were not abundant, the expression of Th17-related genes was increased, and the CD^4+^ cells expressing IL-17A were expanded. Further, bacterial isolates supplemented with *Firmicutes* can eliminate the enhanced Th17 response *in vitro*, and the results support the use of ecotherapy strategies rich in *Firmicutes* to prevent or treat UC.

Populations of T cells that are separated from the colons of mice treated by FMT exhibit a decrease in their ability to proliferate compared to those isolated from colitis mice without FMT treatment. Phenotypically, FMT-treated mice show lower percentages of CD^8+^ T and CD^4+^ T cells, which express the cytotoxicity-related molecule CD107a. This further supports the observation of a decrease in the pro-inflammatory phenotype of colonic T cells in mice treated with FMT (Burrello et al., 2019).

### Inflammatory Cytokines

UC has been linked to abnormal Th2 cell response-mediated inflammation (Oh et al., 2017). Burrello and colleges induced intestinal inflammation in mice using the chronic infusion of dextran sodium sulphate (DSS), which is similar to that observed in patients with IBD. Both the mucus and faeces from normal biological donors were given orally to the mice with established chronic colitis induced by DSS. Normoxic FMT therapy was found to lower the intestinal inflammation, as suggested by a robust decline in not only the colonic expression of the pro-inflammatory marker interferon (IFN)-γ but also tumor necrosis factor (TNF), interleukin (IL)-1β, IL-17, and IL-6. Restoring a main ecology of normal organisms contributed to resolving inflammation (Burrello et al., 2019). Wei et al. (2018) identified that FMT could alleviate the acute colitis stimulated by DSS in mice and FMT results in the upregulation of not only aryl hydrocarbon receptor (AHR) but also transforming growth factor beta (TGF-β) and IL-10 in colon tissues. Wang et al. (2020) carried out a study on active UC patients who received three times FMT from a single donor at an interval of 2–3 months. The clinical response was achieved in 14/16 (87.5%) patients. Compared to those before FMT, serum levels of IL-6, IL-1Ra, epithelial neutrophil activating peptide (ENA)-78, and interferon-inducible protein (IP)-10 significantly decreased after the second FMT (P < 0.05), and serum levels of vascular cell adhesion molecule (VCAM)-1, granulocyte-colony stimulating factor (G-CSF) and mucosae-associated epithelial chemokine (MEC) significantly decreased after both the first and second FMT (P < 0.05). Those findings shed light on the fact that FMT has the potential to control IBD through augmenting anti-inflammation cytokines and reducing pro-inflammatory cytokines.

## Conclusion and Future Perspectives

The understanding of FMT effectiveness for IBD is in its infancy even today. The preliminary findings from medical experiments are conflicting, perhaps owing to the dissimilarities existing in patient populations among various research works, disease seriousness in participants, the delivery processes of FMT, FMT preparations, and the follow-up after transplantation (Rubin, 2015). Nevertheless, the latent potential of FMT for the treatment of IBD should be addressed. It is not clear why some patients with IBD respond impressively following FMT, whereas other patients fail to respond. Nonetheless, it is evident that FMT does not refer to a “one size fits all” and many determinants seem to contribute to its successful use for the treatment of IBD. The host genotype, the course of disease, the use of antibiotics linked to illness onset, the specific types of IBD-linked dysbiosis, and/or donor attributes are all determinants that can potentially dictate the ultimate result. Accordingly, further investigations and a better understanding of this application are required (Rossen et al., 2015). To conclude, applying FMT to manage IBD is likely to constitute an intriguing treatment choice; nonetheless, large and methodical studies are lacking. Further, many concerns related to pathophysiological, methodological, and mechanistic factors require an explanation.

FMT refers to a new potential option for treating IBD; nonetheless, there are many issues that still exist. More research is required to build a more comprehensive and deeper knowledge base, with respect to the entire treatment process ranging from FMT methodologies to FMT immunomodulatory impacts and mechanisms. Being a new treatment for IBD, its efficacy and safety are still not certain, and patient acceptability is also not high; accordingly, longer-term follow-up investigations are required. Standards regarding donor selection are also required. Through further studies, better methodologies and more effective protocols to prepare fecal substances and administer FMT will be realized. Moreover, related laws and regulations need to be formulated to standardise and limit all aspects of the treatment.

## Author Contributions 

PT and XL collected the papers and data, analyzed the conclusions, drafted the manuscript. JS and QF presented the idea of this paper, supported the funding, analyzed the conclusions, drafted, and revised the manuscript.

## Funding

Supported by grants from National Natural Science Foundation of China (No. 81770545 & 81701746) and MDT Project of Clinical Research Innovation Foundation, Renji Hospital, School of Medicine, Shanghai Jiaotong University (PYI-17-003), and North Shanghai Medical Star Talent Training Program of Baoshan Branch Renji Hospital (rbxxrc-2019-008).

## Conflict of Interest

The authors declare that the research was conducted in the absence of any commercial or financial relationships that could be construed as a potential conflict of interest.
